# Spectroscopic OCT by Grating-Based Temporal Correlation Coupled to Optical Spectral Analysis

**DOI:** 10.1155/2008/752340

**Published:** 2008-03-13

**Authors:** L. Froehly, M. Ouadour, L. Furfaro, P. Sandoz, P. Leproux, G. Huss, V. Couderc

**Affiliations:** ^1^Département d'Optique P.M. Duffieux, Institut FEMTO-ST, UMR 6174 CNRS, Université de Franche-Comté, 25030 Besançon Cedex, France; ^2^ Institut de Recherche (XLIM), UMR 6172 CNRS, 123 avenue Albert Thomas, Limoges Cedex 87060, France

## Abstract

Spectroscopic optical coherence tomography (spectroscopic OCT) is an echographic-like optical method for biomedical functional imaging. Current spectroscopic optical coherence tomography (OCT) methods rely on a posteriori numerical calculation. We present an alternative for optically accessing the spectroscopic information in OCT, that is, without postprocessing, by using a grating-based correlation and a wavelength demultiplexing system. Spectrally resolved A-scan is directly recorded on the image sensor. Due to the grating-based system, no correlation scan is necessary. The signal is registered in the wavelength-depth plane on a 2D camera that provides a large number of resolved points. In the frame of this paper, we present the principle of the system as well as demonstration results. Advantages and drawback of this system compared to others are discussed.

## 1. INTRODUCTION

For a decade optical coherence tomography (OCT) shows a growing interest in the field of biomedical imaging. Main reasons are the nondestructive character of these methods, the image resolution down to micrometer scale either in depth or in plane, and also the possibility to make in vivo, nondestructive optical biopsies. Commercial systems are available in the frame of ophthalmology (Zeiss, Optovue, OTI, Topcon, etc.) [1].

There are quite a lot of different configurations to get those optical echographic images [2]. For a few years the need of new contrast agents has grown with the use of OCT as a help in medical diagnosis. Then new functionalities in OCT systems have been investigated. They are mainly as follows:

- polarization OCT imaging [3] accesses tissues properties (structural organization),
- spectroscopic OCT [4] accesses in vivo local tissue (or cell) absorption that informs on cell metabolism or molecule disorders,
- CARS-OCT [5] accesses tissues spectral features in the range of Raman spectrum (molecules vibrational spectra).

Existing spectroscopic OCT systems are mainly based on postnumerical processing of OCT signals acquired in the time domain or in the frequency domain. Works have recently been performed which demonstrate optically processed spectroscopic OCT images [4, 6] in the frame of classical time domain OCT using wavelength demultiplexing.

This paper presents an alternative system which accesses optically the spectrally resolved tomographical information in depth. The presented system is a hybrid system in the depth-wavelength plane. It does not require any moving part nor numeric signal processing. Then the spectrotomogram acquisition rate is the detector frame rate since the signal processing is realized optically.

We demonstrate that this kind of system allows TD-OCT without moving part. This capability was already reported by Hauger by using a “Young-like” correlation system [9]. However, in Hauger's scheme, the scan depth capacity is necessarily restricted due to the number of pixels required for resolving fringe patterns. In our case, the use of a diffraction grating as temporal correlator enhances the measurement range achievable with a given number of pixels. The inherent principle is similar to the one that has been first introduced in 1957 for spectroscopy [10]. This method was adapted more recently to numerous tomographic devices [11–16].

In this paper, we describe completely the working principle of the proposed system. Experimental results validate the system principle and are compared successfully with simulations. The present system specifications are given while there are subject to significant improvements by optimizing the different elements. Finally, advantages and drawbacks of this new approach are discussed with respect to existing spectroscopic OCT systems.

## 2. SYSTEM PRINCIPLE

### 2.1. Experimental setup

**Figure 1:**
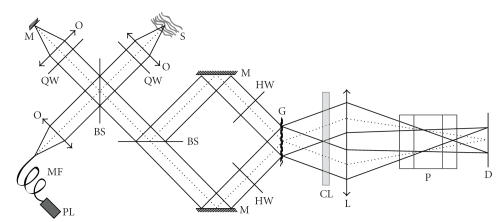
Setup for optical spectrotomography: M: mirrors; BS: polarizing beam-splitter cube; QW: quarter wave plate; HW: half-wave plate; L: spherical lens; CL: cylindrical lens; PL: supercontinuum light source; D: CCD detector; G: transmission diffraction grating 528 gr/mm; P: removable direct vision prism; O: microscope objectives (4×magnification); S: sample.

The experimental setup used is composed of three main parts as depicted in Figure 1. Firstly, the sample information is encoded via a Linnik interferometer. The latter is illuminated with a supercontinuum of light issued from a microstructured optical fiber pumped by a Q-switched Nd-YAG laser [17]. The supercontinuum has a full spectral range from 350 nm up to 1700 nm. However, in experiments reported here, the effective bandwidth is of 100 nm centered around 550 nm. This reduction that could be less restrictive results in practice from the limited size of components of the output spectroscope described below. The average power incident onto the sample is about 500 *μ*W. Since this low coherence light source has a transversally single-mode emission, the sample is illuminated only along the axial point spread function of the microscope objective used. Therefore, the OCT information finally obtained with depth-wavelength resolution corresponds also to this sample volume.

Secondly, the light beams issued from the Linnik interferometer are directed toward a second interferometer. In this Mach-Zehnder-like interferometer the output beam-splitter is replaced by a transmission diffraction grating disposed in the perpendicular direction. Because of the incident angles of the two beams, the transverse direction of the diffraction grating introduces a time-delay varying linearly between the recombined beams. The sample depth is thus encoded across the grating that forms a time correlation axis. A key property of that configuration is to reduce significantly the carrier frequency of the interference fringes. This effect can be explained by the change of the average propagating direction of the interfering wavefronts after diffraction in the −1 order.

The third part of the setup is primarily an imaging system that forms the image of the diffraction grating on a two-dimensional CCD image sensor (8 bits, 1024 × 768 pixels, frame rate 29 Hz). Then the lines of the CCD camera encode the depth of the sample and an A-scan is obtained without scanning along the image lines. Since light incident on the diffraction grating is issued from the same sample line, the different lines of the recorded image carry the same depth information. In a first step, we do not consider the cylindrical lens and the prism that concern spectroscopic displaying as discussed below.

The cascade of the two interferometers should result in autocorrelation. In practice, interfering beams are cross-polarized in both interferometers thanks to polarization multiplexing (quarter and half waveplates represented in Figure 1). Thus we perform intercorrelation instead of autocorrelation.

**Figure 2:**
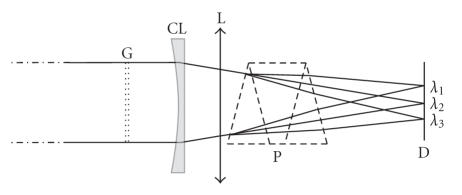
Side view of the spectroscopic output imaging system: G: diffraction grating; CL: cylindrical lens; L: spherical lens; P: prism; D: detector.

Complementary elements are necessary for obtaining spectrally resolved tomograms. These optical elements are the prism (P) and the cylindrical lens (CL) that affect the light propagation only along the vertical direction, that is, perpendicularly to the plane of Figure 1. Then the output imaging system becomes a spectroscope as it is clearly visible on Figure 2, which represents a side view of the output imaging system. Thus the vertical direction of the CCD camera becomes a spectral axis. In this spectroscopic configuration, each image line is associated to a particular wavelength and is illuminated by a restricted bandwidth. The width of the latter determines the spectral resolution that is easily tuned through the position of the cylindrical lens (or its focal length). In this way, each image line provides a band-limited A-scan and the whole image forms a spectrotomogram.

### 2.2. Theoretical analysis of recorded signals

**Figure 3:**
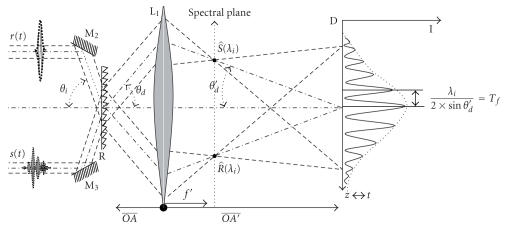
Parameters for correlator working principle.

Let us again consider the output imaging system without the spectroscopic elements for describing temporal correlation using a diffraction grating. The principle is depicted in Figure 3. The lens L1 images the grating plane on the CCD detector plane D. Then we have $\bar{OA^{\prime }}/\bar{OA}$ = *γ* ≈ *θ_d_*/*θ′_d_* in the case of small angle approximation where *γ* is the magnification factor. This assumption is valid in our experiment since the size of the detector is much smaller than the distances of the magnification system (ratio 1.100).

Two temporal signals *r*(*t*) and *s*(*t*) with the same polarization state are incident onto the grating (*R*) with opposite angles *θ_i_* and −*θ_i_*. *r*(*t*) and *s*(*t*) have complex spectrum given by $\hat{R}$(*ν*) and $\hat{S}$(*ν*), respectively, where $\hat{R}$(*ν*) and $\hat{S}$(*ν*) are Fourier transforms of *r*(*t*) and *s*(*t*). $\hat{R}$(*ν*) and $\hat{S}$(*ν*) can be expressed with their complex form $\hat{R}$(*ν*) = *R*(*ν*)*e* ^j⋅φR(ν)^ and $\hat{S}$(*ν*) = *S*(*ν*)*e* ^j⋅φR(ν)^. The moduli of these spectra are directly accessible in the spectral plane of the spectrometer which is physically in the back focal plane of the lens L1.

Light at wavelength *λ_i_* coming from *r*(*t*) and *s*(*t*), respectively, focuses in the spectral plane on two points that are symmetrical with respect to the optical axis. Then they produce in the detector plane D a classical two-wave interference pattern of period *λ_i_*/(2 ⋅ sin *θ′_d_*) = *T_f_*, where *θ′_d_* is the diffraction angle at wavelength *λ_i_*. This configuration is similar to Young's experiment with the important difference that the position of the secondary sources is dependent on the wavelength because of the diffraction law. Each wavelength *λ_i_* leads to its own fringe pattern and all these patterns are summed in intensity because of the noncoherence of fringe patterns obtained with different wavelengths. The resulting pattern is the sum over the effective spectral bandwidth of those individual “Young-like” fringe patterns. This effective bandwidth depends on the detector spectral bandwidth, on the size of the lens L1, and on the grating dispersion power.

In the following analysis, we assume that the frequency of a specific fringe pattern does not exhibit any nonlinearity (plane wave approximation). The upper description can then be written as follows to access the signal *C*(*z*) in plane D:

(1) C(z)=I0+∫νS^(ν)⋅R^(ν)¯⋅e(−j((4πν/c)sin⁡θd′)⋅z)dν +∫νR^(ν)⋅S^(ν)¯⋅e(+j((4πν/c)sin⁡θd′)⋅z)dν

where $\bar{\left(\right)}$ denotes here the conjugate of the complex spectrum of the fields whereas in previous section this notation was denoting the algebraic measure. The grating relation is

(2) $$\sin\theta_{d}-\sin\theta_{i}=-\frac{\lambda }{\Lambda }$$

for the minus one diffraction order where Λ is the grating periodicity. Relations (1), (2) together with the magnification relation lead to the following formulation of *C*(*z*):

(3) C(z)=I0+2Re[∫νR^(ν)S^(ν)e−j2π((2z/γc)sin⁡θi)νej(4πz/γΛ)dν],

where *z* is the horizontal coordinate on the CCD camera lines, *I_0_* the background intensity, and *ℛ_e_* designs the real part. The grating effect appears in the term *e* ^*j*(4*πz/γ*Λ)^ that introduces the fringe frequency shift (the reference beam $\hat{R}$(*ν*) is assumed to be real while $\hat{S}$(*ν*) can be either real or complex depending on the optical sample properties). The spatial frequency of correlation fringes is drastically reduced to become compatible with the modulation transfer function of a CCD sensor. This “moiré-like” effect is of particular interest in terms of detection because the carrier frequency could be tuned independently on the envelope (by changing the beam incidence angle onto grating): we then have a two scale system where the carrier has a usual phase change sensitivity of 2*π* per wavelength of optical path change whereas the envelope remains roughly unchanged with a path length change sensitivity of the order of the source coherence length.

Equation (3) shows clearly the correlation operation between the temporal fields realized by the system. The temporal variable is spatially displayed through the variable change.

The introduction of the cylindrical lens and of the direct vision prism can be taken into account easily. Because of the spectroscopic device, each line of the detector receives a limited bandwidth of light that can be expressed through the spectroscope frequency response $\hat{F}$(*x, ν*). Then, the correlation signal depends on the vertical coordinate on the camera and becomes

(4) C(z,x)=I0+2Re[∫νF^(x,ν)R^(ν)S^(ν)e−j2π((2z/γc)sin⁡θi)νej(4πz/γΛ)dν].

## 3. EXPERIMENTAL RESULTS

**Figure 4:**
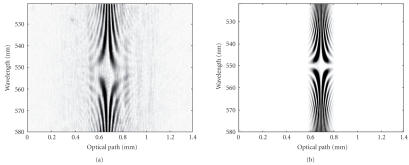
Experimental (a) and simulated (b) spectrotomograms.

Figure 4 shows a comparison between experimental (Figure 4(a)) and theoretical (Figure 4(b)) spectrotomographic signals in the case of a single reflection on a mirror used as a sample (therefore, a single correlation peak is visible). The hyperboloid aspect of these fringe patterns results from grating-based correlation. For the wavelength of 550 nm, the diffracted beams are parallel to the optical axis and the fringe period of the resulting interference pattern is infinite. For other wavelengths, the fringe period increases progressively as the wavelength wanders away from this central wavelength. The shape of this fringe pattern demonstrates clearly the capability of a grating-based correlator to reduce drastically the fringe number,while keeping an important inspection depth. The fringe frequency is no more defined by the incident angles *θ_i_* but by the diffracted angle viewed by the imaging lens L1 *θ′_d_*. Therefore, the pixel number of the camera can be much smaller than that normally given by the Nyquist-Shannon criterion with interference angle *θ_i_*. In our case, the 1024 horizontal pixels of the CCD camera are able to encode an inspected depth larger than 1 mm.This could not be achieved without grating as in the case of Hauger et al. [9].

**Figure 5:**
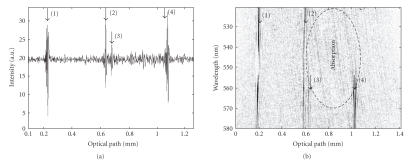
Experimental OCT signals obtained on an eosine solution layer sandwiched by capillarity between two microscope cover slides. (a) Classical “A-scan” obtained with our system (without scanning), (b) instantaneous spectrotomographic signal with visible depth-resolved spectral absorption of the eosine layer.

Figure 4 shows also the tradeoff between spatial and spectral resolution as known in spectroscopic OCT [18] and given by the Fourier relationship *δν* × *δt* = 1. The width of the correlation trace is about 300 *μ*m in the figure and results from a 1 nm spectral resolution. With the proposed setup, a continuous set of band-limited A-scans is recorded simultaneously as presented in Figure 4. The interest of such an optically generated spectroscopic decomposition of A-scans is illustrated in Figure 5 that was obtained on a home-made sample. This sample is composed of two microscope coverslips sandwiching by capillarity a 2% Gifrer Eosine solution. Figure 5(a) is the tomographic signal obtained with this sample without spectral resolution (no wavelength demultiplexing, e.g., without prism and cylindrical lens). Four interfaces are visible (shown with black numbered arrows) corresponding to the four diopters (air-glass, glass-solution, solution-glass, and glass-air). From this recording, we access to the optical thickness of both the coverslips and the eosine solution.

After insertion of the prism (P) and of the cylindrical lens (CL) we obtain the spectrotomogram of Figure 5(b) providing depth-resolved spectroscopic information simultaneously to tomographic information. The vertical axis corresponds to wavelengths and the specific absorption of the eosine solution is clearly visible. The last two diopters are only visible for a fraction of the spectrum, for example, outside the eosine absorption band. The spectral band corresponding to the upper part of the figure is absorbed by the eosine solution and diopters placed behind are not detected. Such a spectrotomogram reveals the spectral behavior of the inspected medium and the absorption band observed is matching that found in the literature [21] (maximum absorption at 520 nm for a PH solution of 4). The wavelength calibration was performed by using 3 interference filters (519 nm, 549 nm, 580 nm) and by taking into account the quadratic spatial dispersion of the prism.

The depth resolution of our system is classically given by the coherence length of the light source viewed by the detector: *l_c_* ∝ λ^2^/Δ λ. We use a 100 nm bandwidth centered on 550 nm. This bandwidth should results in a full depth resolution of 3 *μ*m. It is clearly visible on Figure 5(a) that this resolution is rather 15 *μ*m which is wider than the expected 3 *μ*m one (in fact 6 *μ*m as we realize a correlation operation what involves a widening of the signal envelope). By looking carefully at Figure 5(a), this difference is mainly explained by the residual dispersion of the system that is visible in the fringe period variation of the correlation peaks.

In the case of Figure 5(b), the depth resolution (at 550 nm) is about 25 *μ*m and this results in a spectral resolution of 12 nm. The proposed system enables a simple, continuous, and versatile tunability of the spectral (e.g., axial) resolution by adjusting the position of the dispersive elements. The lateral spatial resolution is classically given by the objective used and has been measured to be about 20 *μ*m (US Air Force Pattern).

## 4. DISCUSSION

### 4.1. System performances

At this stage of system development, signal-to-noise ratio (SNR) or sensitivity measurements are not representative of the ultimate method capabilities since detection elements are not optimized yet. Indeed we use very standard components and we have not yet implemented any heterodyne detection. We have nevertheless perform a measurement of the minimum reflected power we are able to detect from the sample: *P*_min_ = 40 nW, for a reference signal with a power of *P*_ref_ = 600 nW.

However, one may notice already the following points. Our detection scheme differs from TD-OCT ones while reconstructed A-scans are quite similar. On the one hand, no scanning is required so that the integration time on the detector can be much longer and then produces an increase of the SNR. On the other hand, dynamics and noise performances of image sensors are usually worse than those of photodiodes or PMTs. Finally, our detection is closer to FD-OCT ones since each A-scan information is contained on one CCD line. A fundamental difference with FD-OCT is that the signal contrast (fringe contrast of the channeled spectrum) could be quite high in FD-OCT even in a case of several interfaces. Our system suffers the same drawback than TD-OCT: when the number of interfaces to resolve increases the contrast of the interference pattern decreases, what also leads to a decreasing of the SNR. This is due to the the sum in intensity of monochromatic interference patterns which results in a strong DC part. To improve the SNR of our system as well as to remove this DC part which lower the detection dynamic, a CMOS detector with on-pixel signal processing capability will be used. This detector, called smart pixel detector array (SPDA), has already proven to be efficient in parallel TD-OCT [19]. We are currently working on the adaptation of this system to our grating-based correlation device.

### 4.2. Numerical versus optical processing

Numerically processed spectroscopic OCT has already proven to be quite efficient for accessing depth resolved information [20] in “real-time.” So one could ask if the method proposed in the frame of this paper does present any interest in some cases.

Let us first consider the speed argument: the real time capability of numerically processed spectroscopic OCT depends on processor speed. Indeed the computation time necessary to process a digital Fourier transform and extract its modulus and phase determines the speed of such systems. Computation capabilities are known to improve continuously year after year. As an example in 2004 a current 2.4 GHz Pentium IV processor would have calculated a fast Fourier transform (FFT) of a 1024 point array in 20 milliseconds (with modulus and phase extracted). In 2007 an Intel Core 2 at 2.33 GHz will do the same computation in only 2 milliseconds. Then there is a speed gain of 10 in 3 years. A classical image acquisition board in 2004 proposed a sample rate of 5 M sample/s; what allowed a 1024 point array acquisition in only 200 microseconds (a gain of a factor of 100 with respect to the FFT computation time). In 2007, this sample rate has also increased and is now about 50 M sample/s. The gain is then about the same than for FFT computation time. This means that the same technological progress, that provides us with ever-increasing computation speeds, also permits increased data acquisition rates. Then it is clear that computation time remains the bottle neck of methods based on numeric processing of A-scans. In this context, the development of an all-optical method is obviously of scientific interest.

The second argument is the number of resolved points in the wavelength-depth space. In the case of our system, the number of resolved points is directly related to the total number of pixels of the two-dimensional detector. To access the same resolution in TD-OCT, a much higher number of acquired points will be required, which will slow down the acquisition rate. It is even worth in FD-OCT as the Fourier transform of the spectrum gives rise to three terms: an autocorrelation term, the temporal term of interest, and its complex conjugate. This means that the depth resolution is at least divided by a factor of two.

Let us take an example: in time domain OCT the best delay line could reach a depth range of 3 mm at a repetition rate of 2 kHz [22]. If we consider a coherence length of the light source of 1 *μ*m (quite current in high-resolution OCT) this means that the number of points to be resolved is about 3000. According to Nyquist-Shannon criteria, at least 12000 points have to be acquired at a central wavelength of 1 *μ*m (24000 at 0.5 *μ*m). At a repetition rate of 2 kHz, this means a sample rate of at least 24 M sample/s (resp., 48 M sample/s). This speed could be achieved with current acquisition boards but we clearly see a strong dependence of the system performance on the acquisition rate of the acquisition boards.

The case of spectroscopic Fourier domain OCT is even worth since the number of effective pixels is at least divided by two because of the Fourier transform operation (if the autocorrelation term has been removed by phase shifting technique). This means that for such an acquisition, a linear detector with 24000 to 48000 pixels would be needed. The case of swept source OCT is about the same. Indeed the need of FFT is the same than for Fourier domain OCT so that the number of acquisition points is also the same. This means that 24000 to 48000 acquired points would also be necessary. To acquire such a number of points at 50 M sample/s assumes a wavelength sweeping repetition rate of 1 kHz. This has been recently achieved (up to 370 kHz) with new swept laser sources [23]. But the remaining problem is the FFT computation time which is of the order of 20 milliseconds (for 50000 points to be acquired) and that slows down the spectrally resolved A-scan rate to 50 Hz. If we now consider our system, taking into account the same Nyquist-Shannon criteria, 256 × 192 = 49152 points are accessed with a standard CCD of 1024 × 768. This amount is larger than that needed in this example. Indeed a region of interest of 512 × 512 pixels would be sufficient what would benefit to the frame rate which could reach easily 200 Hz with quite classical array detectors. Then the system proposed in this paper is potentially able to acquire spectroscopically resolved A scans at a frequency of 200 Hz with a depth range of 3 mm and a full spectral bandwidth of 400 nm. The resolution in either spaces (depth or wavelength) is only limited by the fundamental Fourier-Heisenberg tradeoff and not by the optical system itself. These considerations demonstrate the scientific interest of studying such an all-optical solution for optical coherence spectrotomography.

### 4.3. Dispersion compensation

In OCT the sample dispersion is normally balanced by adding a cell with a medium of optical properties (refractive index, optical depth) similar to the ones of the sample under test in the reference arm of the interferometer. This “rule of thumb” is still valid while the sample-induced dispersion does not widen the coherence length for more than approximately 4 times the coherence length of the source. When this is no more true a residual depth-dependant dispersion cannot be balanced with this classical technique. Then the resolution as well as the signal-to-noise ratio is suffering for this residual dispersion. The system that we proposed is able to compensate for this linear depth-dependant dispersion. The principle is to use high-order (> 1) dispersion properties of diffraction gratings in a way very similar to what is already known and used in pulse stretching and compression. This aspect is beyond the scope of this paper and will be developed and demonstrated in a forthcoming publication.

## 5. CONCLUSION

We have presented a new system which allows the simultaneous and instantaneous display of a tomographical information resolved over continuous spectral channels. This system does not need any moving part to obtain the spectrally resolved A-scan neither than any numerical processing. The system benefits from a number of depth-wavelength resolved points which is only determined by the total number of pixels of the array detector. Experimental proof of principle has been given and compared successfully to results expected from theoretical developments. The inspection of a home-made absorbing sample demonstrates the ability of our all-optical system to render the in-depth spectral behavior of the inspected medium.

A discussion has been proposed to evaluate advantages and drawbacks of our system. Further steps of our works will be to implement a heterodyne detection using a smart pixel detector array based on CMOS technology. This should improve the SNR of our system as well as the sensitivity as the DC part will be removed. Heterodyne detection is widely known to increase by several orders of magnitude performances of detection systems. Another interesting specificity will also be studied in detail: the capacity to compensate for dispersion with a linear depth-dependant law introduced by a tilt of the correlation grating. This point is indeed of prime importance when speaking about depth resolution of the order of a few *μ*m together with penetration depths into tissues up to the mm range. All these studies are an encouraging scientific basis for developing a system sensitive enough to screen wavelength absorption inside biological tissues on a wide spectral band. This could be applied, we think, for early detection of metabolic disorders inside biological tissues. Indeed spectroscopic analysis is a valuable contrast agent to give information about specific absorption of cancerous tissues especially irrigated with deoxygenated blood at an early stage of their development. This added to the inherent capability of OCT to image inside tissues will allow the simultaneous dimensioning of the lesion depth extension.
